# Lipid profile of *Trichinella papuae* muscle-stage larvae

**DOI:** 10.1038/s41598-020-67297-8

**Published:** 2020-06-23

**Authors:** Suthee Mangmee, Poom Adisakwattana, Phornpimon Tipthara, Nattapon Simanon, Piengchan Sonthayanon, Onrapak Reamtong

**Affiliations:** 1 Department of Molecular Tropical Medicine and Genetics, Faculty of Tropical Medicine, Mahidol University, Bangkok, 10400 Thailand; 2 Department of Helminthology, Faculty of Tropical Medicine, Mahidol University, Bangkok, 10400 Thailand; 3 Mahidol-Oxford Tropical Medicine Research Unit, Faculty of Tropical Medicine, Mahidol University, Bangkok, 10400 Thailand

**Keywords:** Chemical biology, Lipidomics

## Abstract

Outbreaks of trichinellosis caused by *Trichinella papuae* have been reported in South-East Asia. Mebendazole and thiabendazole are the treatments of choice for trichinellosis; however, both drugs result in significant side effects and are less effective for muscle-stage larvae (L1). An alternative therapeutic agent is needed to improve treatment. Information on lipid composition and metabolic pathways may bridge gaps in our knowledge and lead to new antiparasitics. The *T. papuae* L1 lipidome was analysed using a mass spectrometry-based approach, and 403 lipid components were identified. Eight lipid classes were found and glycerophospholipids were dominant, corresponding to 63% of total lipids, of which the glycerolipid DG (20:1[11Z]/22:4[7Z,10Z,13Z,16Z]/0:0) (iso2) was the most abundant. Overall, 57% of *T. papuae* lipids were absent in humans; therefore, lipid metabolism may be dissimilar in the two species. Proteins involved *T. papuae* lipid metabolism were explored using bioinformatics. We found that 4-hydroxybutyrate coenzyme A transferase, uncharacterized protein (A0A0V1MCB5) and ML-domain-containing protein are not present in humans. *T. papuae* glycerophospholipid metabolic and phosphatidylinositol dephosphorylation processes contain several proteins that are dissimilar to those in humans. These findings provide insights into *T. papuae* lipid composition and metabolism, which may facilitate the development of novel trichinellosis treatments.

## Introduction

*Trichinella* is a genus of parasitic roundworms that cause trichinosis, also known as trichinellosis, which infect domestic and sylvatic animals. The number of global outbreaks appears to have sharply increased, reflecting changes in the parasites’ epidemiology^1^. In South-East Asia, outbreaks of trichinellosis caused by *T. papuae* occurred in 2006^2^ and 2007^3^. *T. papuae*, which belongs to the non-encapsulated trichinella clade, lives predominantly in tropical rainforests^4^. Ingestion of raw meat containing parasite cysts leads to infection, and the larvae released from adult females invade host muscles resulting in trichinellosis pathology. Common symptoms are eye puffiness, splinter haemorrhaging, nonspecific gastroenteritis, and muscle pain^5^. Trichinosis treatment is based on anti-inflammatory drugs and anthelmintics, such as mebendazole and albendazole^6^; however, the effectiveness of anthelmintic treatment is an issue of debate. In the treatment of myositis during a trichinellosis outbreak in Thailand, mebendazole and thiabendazole were found to be more efficient than placebo or fluconazole. However, 30% of volunteers could not tolerate the side effects of thiabendazole^7^. In addition, in a trial during an outbreak in Italy, between 3% and 45% of patients had a recurrence of various symptoms after a 10-day mebendazole course^8^. Generally, anthelmintic therapy is only considered effective during the intestinal phase of infection, and the drug has poor drug effectiveness in the muscle phase^9^. To improve the effectiveness of the treatment, an alternative therapeutic agent may need to be developed. Most likely, a combination of two or more drugs with different modes of action will be needed for an adequate cure rate and to delay the development of parasite resistance.

Recently, high-throughput technologies have allowed characterization of the lipid profiles of parasites, including unicellular protists and worms. Specific lipid structures and their metabolic pathways could be targets for the development of novel anthelmintics. In addition, the discovery of a vital enzyme in a metabolic pathway that is absent or significantly different from that in the host is an advantage for novel target identification and drug development^10^. Separation techniques, such as gas chromatography (GC) and liquid chromatography (LC), are coupled with mass spectrometry (MS) for lipid detection and quantification^11^. The lipid profiles of leishmania^12^, *Toxoplasma gondii*^13^ and *Haemonchus contortus*^14^ are examples of successful lipidomic approaches. Non-mammalian lipids have been reported in parasite lipidomes, such as glycerolipids or sphingolipids terminated by α-Gal(1 → 6)β-Gal, which is unique in apicomplexans^15^. Information on lipid composition and lipid pathways has led to several antiparasitic designs. Glycosylphosphatidylinositols of *T. gondii* and *Plasmodium falciparum* were found to be candidates for immunotherapeutic strategies^16,17^. Another phosphatidylcholine metabolism in plasmodium was demonstrated to be a novel drug target^18^. Amphotericin B is an antiparasitic drug which binds to ergosterol, which is present in leishmania membranes and absent in mammals. The amphotericin B-induced membrane pore is responsible for ion leakage, contributing to parasite death^19^. Miltefosine^20^, edelfosine^21^ and sitamaquine^22^ are lipid-like drugs used for leishmaniasis treatment; they affect membrane lipid rafts and accumulate in *L. donovani* through passive diffusion.

In this research, we aimed to profile the lipid components of *T. papuae* larvae using MS-based lipidomics. In addition, proteins relating to lipid metabolism in *T. papuae* were compared with those in humans to explore potential new drug targets. The study was designed to identify drug target candidates and their related pathways.

## Results

### Lipid profile of *T. papuae* larvae

**Figure 1:**
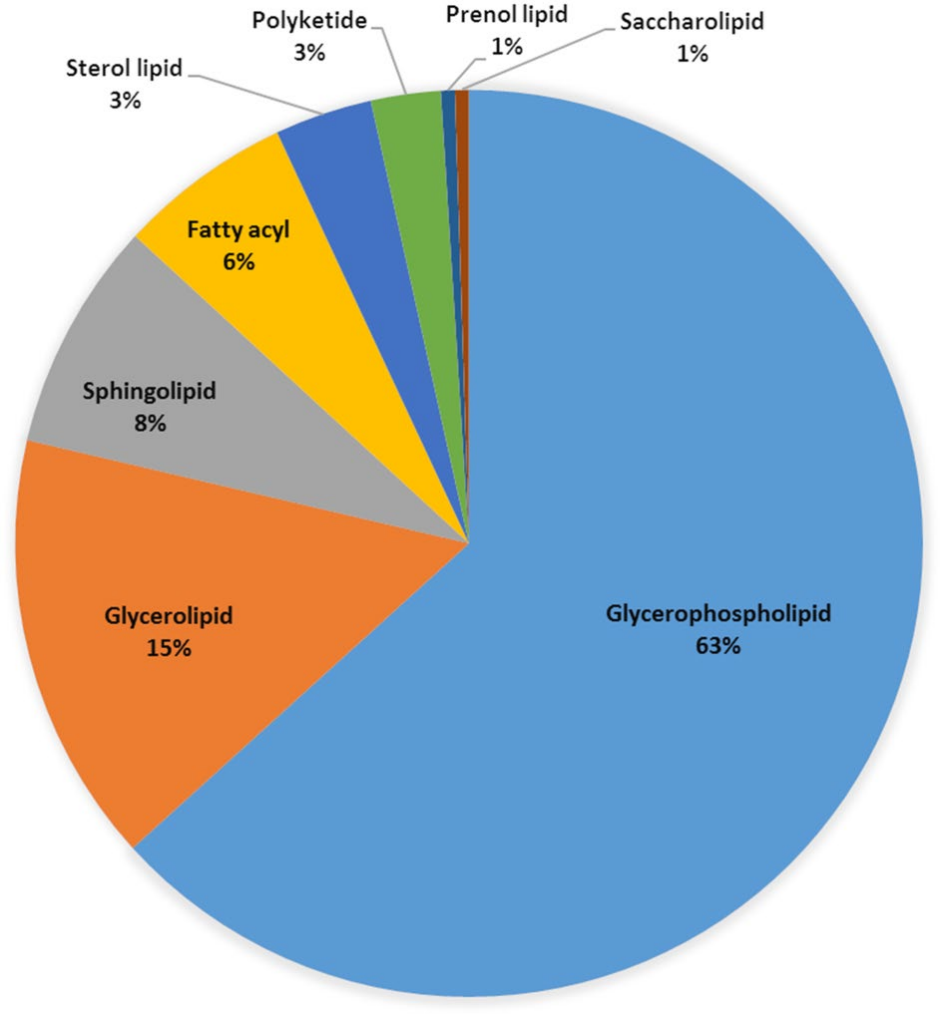
Classification of *T. papuae* muscle-stage larvae lipidome.

Lipid components were extracted from *T. papuae* L1 and analysed by liquid chromatography (LC) coupled with tandem MS (LC-MS/MS). To increase the number of lipids identified, both positive and negative electrospray ionization (ESI) modes were used for MS analysis. A total of 403 lipid components were identified from larval extracts. The positive and negative modes detected 300 and 104 lipid species, respectively (Supplementary Dataset 1). Only a polyketide, ovaliflavanone A, was found by both ionization modes, as shown in Supplementary Fig. 1. Whole lipid components of *T. papuae* L1 were classified into eight classes: glycerophospholipids, glycerolipids, sphingolipids, fatty acyls, sterol lipids, polyketides, prenol lipids and saccharolipids. Glycerophospholipids were the largest component, corresponding to 63% of the total lipids. While glycerolipids and sphingolipids comprised 15% and 8%, respectively. The minor lipid classes were fatty acyl lipids (6%), sterol lipids (3%), polyketides (3%), prenol lipids (1%) and saccharolipids (1%), as presented in Fig. 1. *T. papuae* glycerophospholipids contain fatty acids with carbon chain lengths of 6, 8,12, 13, 15, 16, 17 18, 20, 22, 24 and 26, and 79% of glycerophospholipids contained unsaturated fatty acids. *T. papuae* glycerolipids were found to have fatty acids with carbon chain lengths of 9, 12, 13, 14, 15, 16, 17, 18, 20, 22, 23, 24, 25 and 26, and the majority (87%) also contained unsaturated fatty acids. The sphingolipids were composed of fatty acids with carbon chain lengths of 14, 15, 16, 17, 18, 19, 20, 22, 24 and 26, and 79% of them contained unsaturated fatty acids. Because the glycerophospholipids, glycerolipids, sphingolipids and fatty acyl lipids were the major classes in *T. papuae*, they were further subclassified, as described in Table 1. In the glycerophospholipids, the major lipid subclass was glycerophosphocholine (PC) representing 96 species. While glycerophosphoethanolamine (PE), glycerophosphoserine (PS), glycerophosphoinositol (PI), glycerophosphoglycerol (PG) and glycerophosphate represented 75, 32, 15, 14 and 12 species, respectively. In the glycerolipids, diradylglycerol (DG) was the main subclass, with 27 species. Triradylglycerolipid (TG), digalactosyldiacylglycerol (DGDG), monoradylglycerolipid (MG) and monogalactosyldiacylglycerol (MGDG) described 23, 10, 1 and 1 species, respectively. In the sphingolipids, ceramide was the largest lipid subclass, with 17 species. Sphingomyelin, glycosphingolipid and sulphatide represented 8, 6 and 2 species, respectively. Of the fatty acyl lipids, 15 carnitines were observed, while triacontane and tetraenoic acid each described three respective species.

**Table 1:** Subclasses of fatty acyl, glycerolipid, glycerophospholipid and sphingolipid.

Lipid class	Subclass	No. of components
Fatty acyl	Carnitines	5
	Triacontanes	3
	Tetraenoic acids	3
	Others	14
Glycerolipid	Triradylglycerolipids	23
	Monoradylglycerolipids	1
	Digalactosyldiacylglycerols	10
	Diradylglycerols	27
	Monogalactosyldiacylglycerols	1
Glycerophospholipid	Glycerophosphates	12
	Glycerophosphocholines	96
	Glycerophosphoethanolamines	75
	Glycerophosphoglycerols	14
	Glycerophosphoinositols	15
	Glycerophosphoserines	32
	Others	11
Sphingolipid	Ceramides	17
	Sphingomyelins	8
	Glycosphingolipids	6
	Sulphatides	2

### Comparison of *T. papuae* and human lipidomes

**Figure 2:**
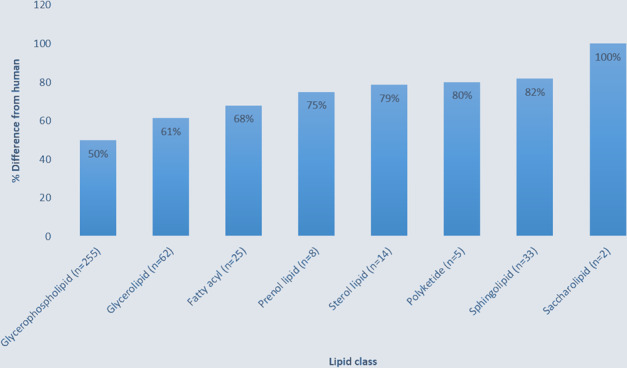
Percentage of *T. papuae* lipids that were different from those in humans.

Each *T. papuae* L1 lipid component was compared to the Human Metabolome Database (HMDB). The HMDB contains the small molecule metabolites reported in humans. The metabolite data has been acquired from different experimental approaches, such as nuclear magnetic resonance (NMR) spectroscopy, gas chromatography–MS (GC-MS) and liquid chromatography–MS (LC-MS)^23^. In *T. papuae* L1, 57% of the total lipids were different from those in humans (Supplementary Dataset 1). All eight lipid classes in *T. papuae* were more than 50% different from human lipids. In particular, saccharolipids were 100% different between *T. papuae* and humans (Fig. 2).

### Quantification of lipid components

**Figure 3:**
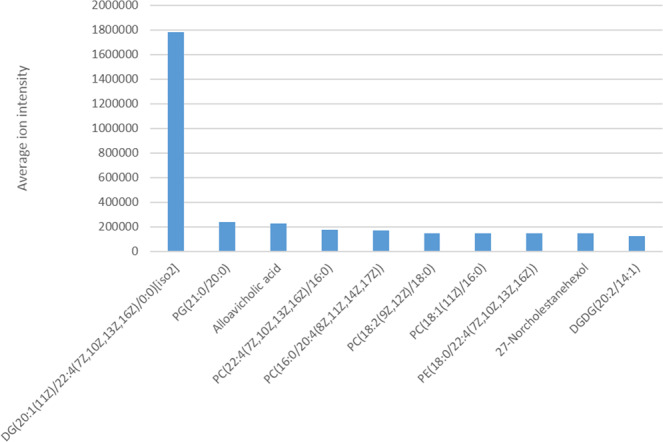
Top-10 most abundant lipids identified in *T. papuae* muscle-stage larvae.

The quantities of each lipid species can be estimated through the ion intensities of LC-MS/MS analysis. The top-10 most abundant lipids are shown in Fig. 3. A glycerolipid, DG (20:1[11Z]/22:4[7Z,10Z,13Z,16Z]/0:0) (iso2), was the most abundant lipid species in *T. papuae* L1. The abundance of this lipid was seven-fold greater than that of PG (21:0/20:0), the second ranked species. The glycerophospholipids, PG (21:0/20:0), PC (22:4[7Z,10Z,13Z,16Z]/16:0), PC (16:0/20:4[8Z,11Z,14Z,17Z]), PC (18:2[9Z,12Z]/18:0), PC (18:1[11Z]/16:0) and PE (18:0/22:4[7Z,10Z,13Z,16Z]), were included in the top ranking lipids after DG (20:1[11Z]/22:4[7Z,10Z,13Z,16Z]/0:0) in *T. papuae* L1. While, sterol lipids, e.g., alloavicholic acid and 27-norcholestanehexol, were also highly abundant. However, the total ion abundance of a specific lipid species doesn’t necessarily indicate this lipid amount since ion abundance was affected by both ionization efficiency and lipid concentration. The different lipid class has quite different ionization efficiency. Therefore, relative ion abundance of lipids from the same class were more appropriate to consider. The top-10 most abundant lipids in each class in are provided in Supplementary Table 1. The most abundant glycerophospholipids were PG (21:0/20:0), PC (22:4[7Z,10Z,13Z,16Z]/16:0) and PC (16:0/20:4[8Z,11Z,14Z,17Z]). Whereas, DG (20:1[11Z]/22:4[7Z,10Z,13Z,16Z]/0:0) (iso2), DGDG (20:2/14:1) and TG (12:0/13:0/15:1[9Z]) (iso6) were the most abundant in the glycerolipid class. The top sphingolipids were SM (d18:1/18:0), SM (d18:1/16:0) and Cer (d14:1/22:0). The fatty acyl lipids with the high yields were 4,8,16-trimethyldotriacontane, 11,21-dimethylheptatriacontane and (9Z)-3-hydroxyoctadecenoylcarnitine. With regards sterol lipids, alloavicholic acid, 27-norcholestanehexol and beta-chlorogenin were the most common. Whereas, the most abundant polyketide species were 4,5-di-O-methyl-8-prenylafzelechin-4beta-ol and ovaliflavanone A. Prenol lipids with high abundance were 35-aminobacteriohopane-32,33,34-triol, coenzyme Q10 and hemigossypol. Of the saccharolipids, butyl 4′-O-butanoyl-6-O-hexadecanoyl-neohesperidoside and DAT (18:0/22:0[2Me{S},4Me{S}]) were the most abundant species. The chemical structures of the most abundant lipids in every class were distinct from those of human lipids. This finding suggests that the lipidome of *T. papuae* L1 is distinct from that of *Homo sapiens*.

### *T. papuae* lipid mechanisms

**Figure 4:**
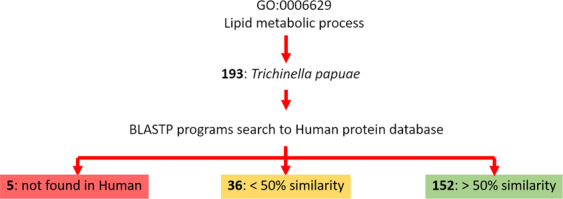
A bioinformatics pipeline for investigating proteins involved in lipid metabolic processes of *T. papuae*.

**Figure 5:**
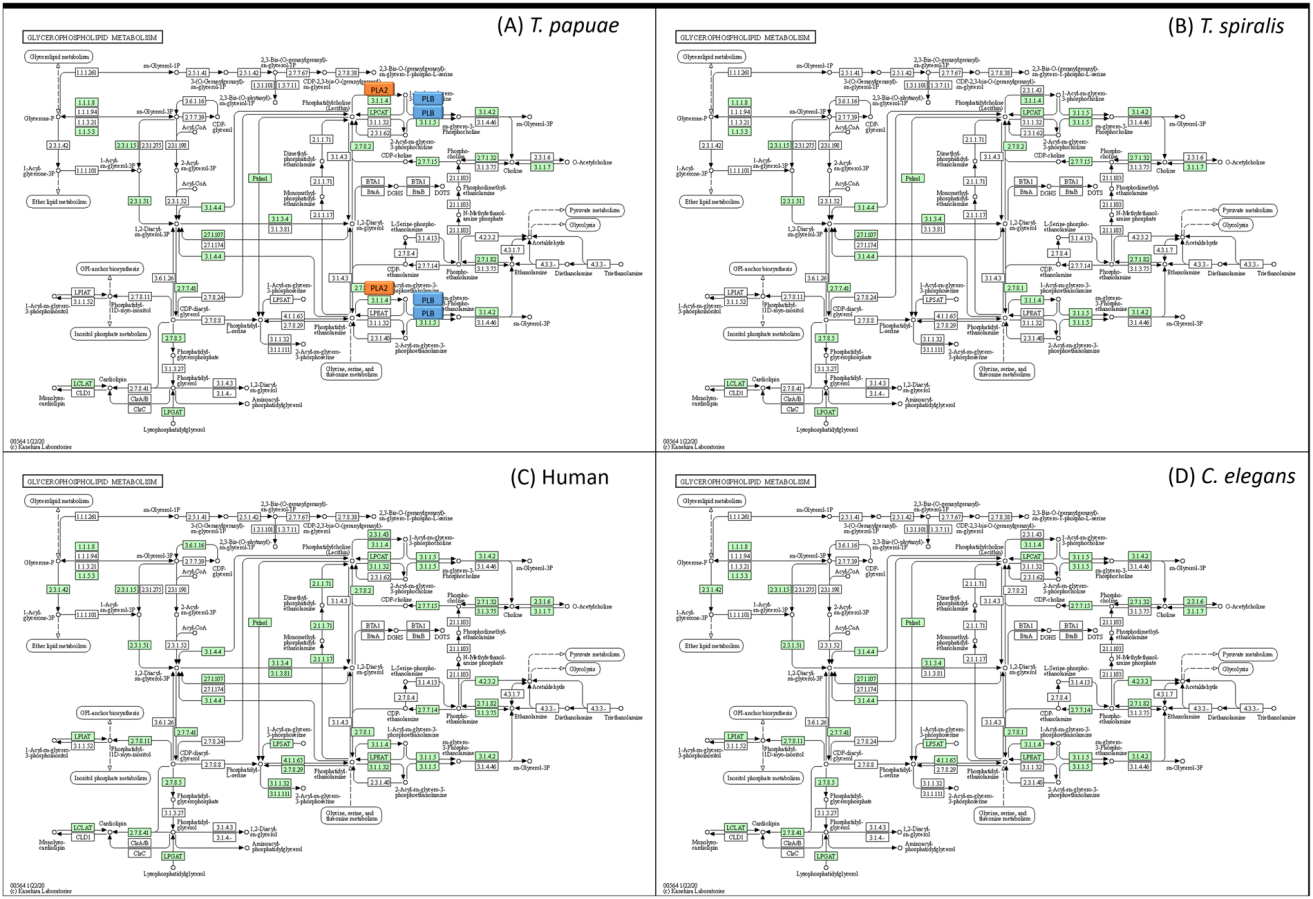
Glycerophospholipid metabolic process of *T. papuae* (**A**), *T. spiralis* (**B**), human (**C**), *C. elegans* (**D**) The orange and blue boxes represent PLA2 and PLB, respectively. The green boxes represent proteins presenting in each organism.

**Figure 6:**
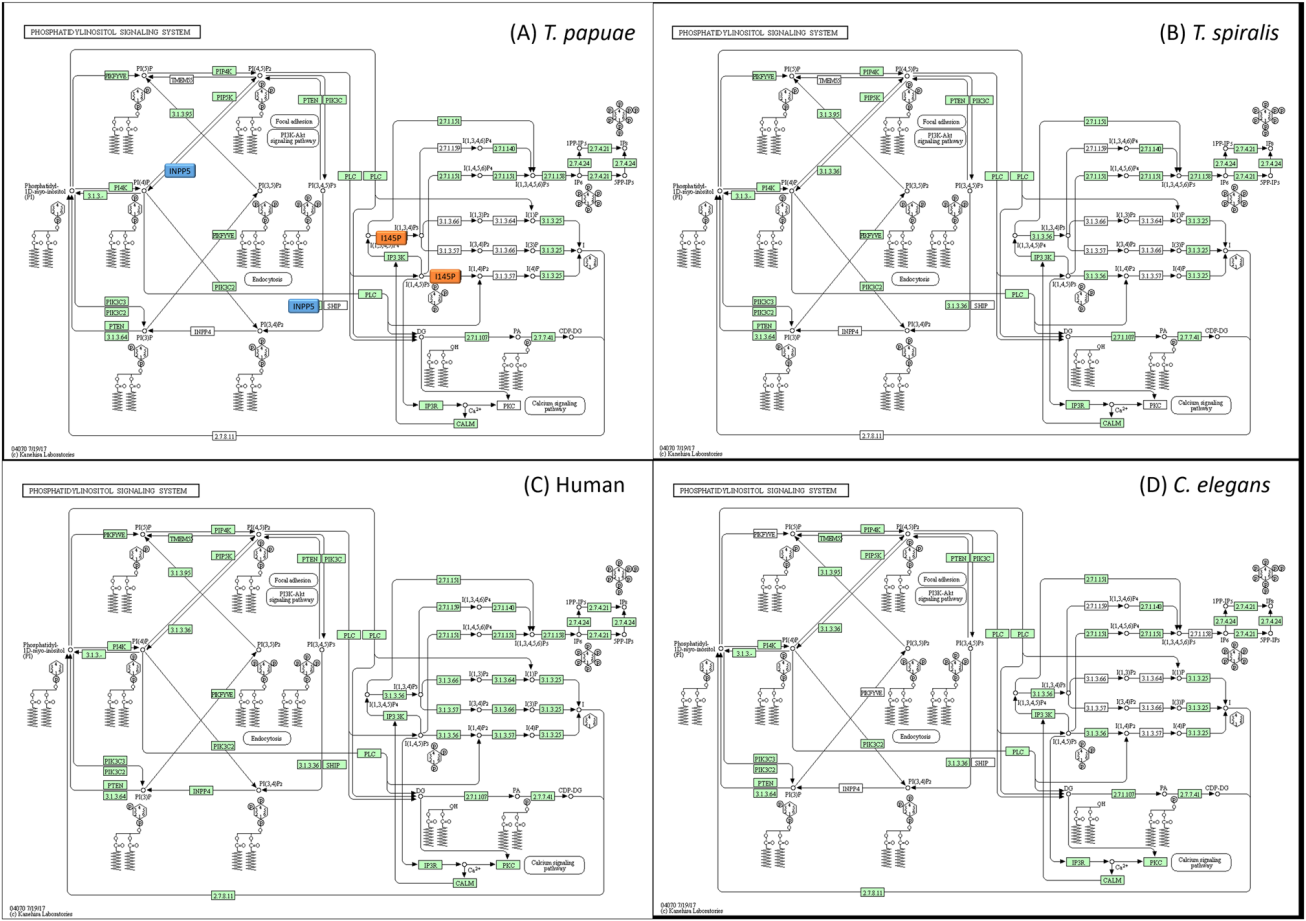
Phosphatidylinositol dephosphorylation process *T. papuae* (**A**), *T. spiralis* (**B**), human (**C**), *C. elegans* (**D**) The orange and blue boxes represent I145P and INPP5, respectively. The green boxes represent proteins presenting in each organism.

Because of the distinct structures of *T. papuae* L1 lipids, their metabolism and synthesis mechanisms may also differ from those of humans. To investigate the proteins involved in the lipid metabolic processes in *T. papuae*, a bioinformatics pipeline was designed, which is shown in Fig. 4. Multiple gene ontology (GO) can be used to annotate the function of single genes; therefore, all genes involved in *T. papuae* lipid metabolism were collated using QuickGO, a web-based tool for GO searching. This annotation program provides GO annotations to proteins in the UniProt Knowledgebase (UniProtKB), RNA molecules from RNACentral and protein complexes from the Complex Portal. The GO:0006629 pathway was used to search for lipid metabolism process genes and 193 gene products were found that belonged to *T. papuae*. All protein sequences involved in *T. papuae* lipid metabolic processes were subjected to the Basic Local Alignment Search Tool (BLAST) to compare their similarity to *T. spiralis*, *T. britovi*, *T. native*, *T. pseudospiralis, C. elegans* and human protein sequences. We found 41 *T. papuae* proteins containing less than 50% sequence similarity to human proteins (Supplementary Table 2 and Supplementary Dataset 2), providing evidence that some cellular lipid processes of *T. papuae* may be dissimilar to those in human. *T. spiralis*, *T. britovi*, *T. native* and *T. pseudospiralis* demonstrated high similarity to *T. papuae* proteins. Whereas, *C. elegans* protein sequences were slightly different from *Trichinella* species. Compared with human proteins, *T. papuae* 4-hydroxybutyrate coenzyme A transferase (A0A0V1M4U9), uncharacterized protein (A0A0V1MCB5) and ML domain-containing protein (A0A0V1MEM4) had the lowest sequence similarity. *T. papuae* 4-hydroxybutyrate coenzyme A transferase is involved in the acetate metabolic process (GO:0006083), propionate metabolic process and methylcitrate cycle (GO:0019679). Whereas, the uncharacterized protein and ML domain-containing protein are associated with the ganglioside catabolic process (GO:0006689) and sphingolipid metabolic process (GO:0006665), respectively. These three proteins are putative candidates for future anti-trichinella drug development. The entire 41 *T. papuae* proteins with low sequence similarity to human proteins involved in lipid metabolism were classified into the 16 lipid pathways shown in Table 2. The major pathways were the glycerophospholipid metabolic process (GO:0006650, 6 proteins), phosphatidylinositol dephosphorylation (GO:0046856, 6 proteins), acetate metabolic process (GO:0006083, 3 proteins), ganglioside catabolic process (GO:0006689, 3 proteins), glycosylphosphatidylinositol (GPI)-anchor biosynthetic process (GO:0006506, 3 proteins) and sphingolipid metabolic process (GO:0006665, 3 proteins). *T. papuae* glycerophospholipid metabolic and phosphatidylinositol dephosphorylation processes present the most attractive targets for drug design because they contain several proteins that are dissimilar to equivalent proteins in humans. These include phospholipase B-like (PLB) and 85/88 kDa calcium-independent phospholipase A2 (PLA2) of the glycerophospholipid metabolic process (GO:0006650, 6 proteins) (Fig. 5A) and type I inositol 1,4,5-trisphosphate 5-phosphatase (I145P), inositol polyphosphate 5-phosphatase K (INPP5) and 72 kDa inositol polyphosphate 5-phosphatase of the phosphatidylinositol dephosphorylation process (Fig. 6A). In addition, a total 15,304 protein sequences of *T. papuae* was collected from UniProt database and analyzed by blast2GO software to match the *T. papuae* proteins into the glycerophospholipid metabolism process and phosphatidylinositol dephosphorylation process as shown in Figs 5A and 6A, respectively. Moreover, the *T. spiralis*, *C. elegan*s and human glycerophospholipid metabolism process and phosphatidylinositol dephosphorylation process are also demonstrated for comparison in Figs 5 and 6, respectively. The green and white boxes are proteins which present and absent in each organism. Due to these finding, proteins in both processes are similar between *T. papuae* and *T. spiralis*. They are slightly different between *C. elegans* and *Trichinella*. While, the proteins in the pathways are distinguishable between nematode and human.

**Table 2:** GO term classification of *T. papuae* proteins involved in lipid metabolic processes.

Pathway	No. of proteins
Glycerophospholipid metabolic process [GO:0006650]	6
Phosphatidylinositol dephosphorylation [GO:0046856]	6
Acetate metabolic process [GO:0006083]; propionate metabolic process, methylcitrate cycle [GO:0019679]	3
Ganglioside catabolic process [GO:0006689]	3
GPI-anchor biosynthetic process [GO:0006506]	3
Sphingolipid metabolic process [GO:0006665]	3
Arachidonic acid secretion [GO:0050482]; lipid catabolic process [GO:0016042]; phospholipid metabolic process [GO:0006644]	2
cholesterol metabolic process [GO:0008203]; lipid catabolic process [GO:0016042]	2
fatty acid biosynthetic process [GO:0006633]	2
lipid biosynthetic process [GO:0008610]	2
lipid metabolic process [GO:0006629]	2
phosphatidylinositol-mediated signalling [GO:0048015]; phosphatidylinositol phosphorylation [GO:0046854]	2
no data	2
angiogenesis [GO:0001525]; dolichol biosynthetic process [GO:0019408]	1
farnesyl diphosphate biosynthetic process [GO:0045337]; geranyl diphosphate biosynthetic process [GO:0033384]	1
steroid biosynthetic process [GO:0006694]	1

## Discussion

According to our LC-MS/MS lipid analyses, both the negative and positive ESI ionization modes increased the number of identified lipids. Some sodium adducts were observed on *T. papuae* polar and non-polar lipids. Cation adducts form easily on the dipoles of lipids in the positive ionization mode^24^. In the negative mode, *T. papuae* PE, PI, PS, sulphatide and non-esterified free fatty acids were detected. These lipid species are acidic lipids containing ionic bonds and were simply deprotonated and ionized as negative ion species. *T. papuae* PC and SM were also detected in the negative ionization mode as a result of their strong zwitterionic structures which can form anionic adducts with small anions^25^.

In the lipid classification experiment, glycerophospholipids (also called phosphoglycerides) were identified as the major lipid class of *T. papuae*. Glycerophospholipids are fatty acid diglycerides with a phosphatidyl ester attached to the terminal carbon. Their abundance is likely to be related to the fact that glycerophospholipids, sphingolipids and sterols are the major constituents of membrane bilayers in eukaryotic cells, with glycerophospholipids being the most abundant^26^. A high percentage of glycerophospholipids has been observed in other parasites, such as *Trypanosoma brucei*^27^ and *S. mansoni*^28^. The *T. papuae* glycerophospholipids were sub-classified into PC (39%), PE (31%), PS (13%), PI (6%), PG (6%) and glycerophosphate (5%). *Onchcerca ochengi*, a filarial nematode, was found to contain PC (57.6%), PE (25.2%), PI (8.5%) and PS (7.8%)^29^. The glycerophospholipids of the two nematodes appear to have similar concentration trends. A study of *S. mansoni* glycerophospholipids reported contents of PC (28%), PE (25%), PS (15%), and PG (8%)^30^. The percentages of PC in *T. papuae* and *O. ochengia* are higher than that in *S. mansoni*, which may be related to their different body walls structures. The outer body wall of *S. mansoni* is a tegument of carbohydrate-containing macromolecules known as the glycocalyx^31^. In contrast, the body wall surface of *T. papuae* is coated with an extracuticular layer of lipids comprised of an abundance of PC^32^. PC is composed of a choline head group and glycerophosphoric acid with a variety of fatty acids, and it has roles not only in biological membranes but also in strategies by which parasitic helminths evade host immune responses. PC attached to a secreted glycoprotein ES-62 of the rodent filarial nematode *Acanthocheilonema viteae* is thought to be responsible for modulating host cytokine production^33^. PC can induce MyD88 protein expression leading to suppression of toll-like receptor 4 (TLR4) and interleukin-33 (IL-33) signalling^34^. Synthetic PC analogues of *A. viteae* reproduce many anti-inflammatory effects and have potential uses as anti-inflammatory drugs^35^. Furthermore, the PC components of *T. papuae* membranes may also play roles in host immune modulation. PE and PS contain phosphorylethanolamine and phosphoserine, respectively, at glycerol substitution sites and many different combinations of fatty acids can attach at the C-1 and C-2 positions of the glycerol. In addition, PEs and PSs are involved in cell membrane metabolism^36^. Oxidized PE and PS promote or suppress human inflammatory phenotypes in monocytes and myeloid dendritic cells, and they promote an inflammatory response by increasing tumour necrosis factor alpha (TNF-α) expression. However, stimulation of monocytes with oxidized PE and PS in the presence of lipopolysaccharides (LPS) results in a decrease in TNF-α and IL-1β^37^. Oxidized PEs and PSs have also been observed in *T. papuae*, and they may be involved in modulating the host inflammatory response. Furthermore, they are possible drug development candidates for human diseases related to oxidative stress and inflammation. One such glycerophospholipid class molecule in *T. papuae* is PI; it contains a glycerol molecule with phosphoinositide and various fatty acids and is a core component of GPI anchors. The GPI consists of PI, glycans and a terminal phosphoethanolamine linked to the C-terminus of the protein. The GPIs of *Plasmodium*, *Trypanosoma*, *Leishmania* and *Toxoplasma* are distinguishable from mammalian GPIs^38^. The GPI-anchoring surface antigens are involved in the pathogenicity of parasitic diseases by causing inflammatory responses and other symptoms, for example hypoglycaemia, acidosis and anaemia^39^. GPI-based vaccines have been demonstrated to reduce parasitaemia and malaria severity^40^. In addition, GPI-related pathways have been previously studied for antiparasitic purposes, e.g., for immune therapeutics and novel drug design^41–43^. There are differences in the structural details of PIs between *T. papuae* and humans, such as oxidization and fatty acid composition. Therefore, *T. papuae* PIs and related biological mechanisms might be useful areas for anti-trichinella research.

With regards to glycerolipids, their structures are composed of a glycerol condensed with one, two, or three fatty acids, yielding MG, DG and TG, respectively. In most eukaryotes, glycerolipids are mainly involved in lipid storage. In *Toxoplasma*, TG is de-novo synthesized through an acyl‐CoA:diacylglycerol acyltransferase‐mediated pathway localized to the parasite endoplasmic reticulum^44^. DGAT (acyl-CoA: diacylglycerol acyltransferase) is a transmembrane enzyme that is thought be the rate-limiting enzyme for glycolipid synthesis. DGAT also presents in *T. papuae* as a hypothetical protein T10_12081 (GenBank: KRZ77819.1). The *T. papuae* protein showed 72% sequence similarity to the diacylglycerol O-acyltransferase of *Brachionus plicatilis* (data not shown). For that reason, *T. papuae* may synthesize glycerolipids through a similar pathway to *B. plicatilis*, and *T. papuae* DGAT may be a good candidate for drug development. Galactolipids, such as MGDG and DGDG, are also members of the glycerolipid class. Interestingly, the MGDGs and DGDGs identified in *T. papuae* were completely distinct from the human galactolipids. DGDGs in *P. falciparum* and *T. gondii* are also different from those in humans, but they show similar properties to plant DGDGs analysed by chromatographic lipid separation^45^. A thorough examination of the galactolipid biosynthesis pathway of *T. papuae* may provide further insights into lipid metabolism in this parasite.

The backbone of sphingolipid is a sphingoid (long-chain) base, which is usually linked to a fatty acid via an amide bond. Sphingolipids are important for cell division, differentiation, cell death, and cell recognition and signalling, and sphingolipid biosynthesis pathways have been proposed as promising targets for therapeutic intervention^46^. A key step in the synthesis of the complex sphingolipids is the glucosylation of ceramide, which is mediated by ceramide glucosyltransferase (CGT). CGT of *C. elegans* has been reported to have roles in oocyte formation and early embryonic cell division^47^. A CGT inhibitor, 1-phenyl-2-palmitoylamino-3-morpholino-1-propanol, was able to reduce cyst transformation of *Giardia lamblia* by 90%^48^. In *T. papuae*, CGT also presents as a ceramide glucosyltransferase-B (GenBank: KRZ80132.1), and it could represent a drug target for trichinellosis treatment. The schistosomal sphingolipids induce T helper 2 cell responses in helminth infections through T cell stimulation^49^. A structural analogue of sphingosine, 2-amino-2[2-(4-octylphenyl)ethyl]-1,3-propanediol (fingolimod), is a new generation of immunomodulator for several autoimmune diseases and has recently completed a phase II clinical trial for sclerosis^50^. Sphingolipids may be involved in *T. papuae* cellular processes and may also act as anti-inflammatory molecules during host immune evasion.

Sterols are necessary for cell membrane structure and function and act as precursors for several steroid hormones. Sterols are generated from acetyl-CoA via a multistep metabolic pathway that includes the key steps acetoacetyl-CoA thiolase, 3-hydroxy-3-methylglutaryl-coenzyme A (HMG-CoA) synthase and HMG-CoA reductase. These three enzymes are expressed in *T. papuae* as 3-ketoacyl-CoA thiolase (GenBank: KRZ75105.1), hydroxymethylglutaryl-CoA synthase 1 (GenBank: KRZ69053.1) and HMG-CoA reductase (GenBank: KRZ76401.1). Statin is one of the main classes of sterol biosynthesis inhibitors, which inhibit HMG-CoA reductase and have been widely used for cholesterol reduction in humans^51^. In *T. cruzi*, inhibitors of HMG-CoA reductase, such as mevinolin (lovastatin), reduced the growth rate of amastigotes. Furthermore, a combination of mevinolin, terbinafine and ketoconazole showed a synergistic effect on amastigotes^52^. Therefore, interference of the *T. papuae* sterol biosynthesis pathway is a potential approach for anti-trichinella development.

Neutral-lipid and glycolipid uptake have been observed in parasitic nematodes, and, although the actual mechanism of lipid absorption has not been investigated, it is likely to be a form of diffusion^53^. Because of the chemical properties of lipids, they must be transported through aqueous environments by other molecules. There are two main types of lipid binding proteins (LBPs) found in nematodes; one carries lipids through aqueous compartments and the other transports lipids across cell membranes. LBPs may also be involved in cellular functions, nutrient acquisition, host–parasite interactions and pathogenesis^54^. Long-chain fatty acid transport protein 1 (GenBank: KRZ68592.1) and fatty acid-binding-like protein 6 (GenBank: KRZ72130.1) were found in *T. papuae* and may play roles in lipid uptake or transportation. In addition, lipid metabolic processes are important for parasitic nematodes. In our study, three *T. papuae* lipid metabolic proteins, 4-hydroxybutyrate coenzyme A transferase, uncharacterized protein (A0A0V1MCB5) and ML domain-containing protein, are conserved among *Trichinella* species and not found in humans. In *T. papuae*, 4-hydroxybutyrate coenzyme A transferase takes part in the fermentation of 4-aminobutyrate to ammonia, acetate and butyrate and has been found in anaerobic bacteria such as *Clostridium aminobutyricum* and *Porphyromonas gingivalis*^55^. *T. papuae* juveniles within nurse cells use facultative anaerobic metabolism^56^, thus, they may use this enzyme for the same purpose as anaerobic bacteria. There is no published information on uncharacterized protein (A0A0V1MCB5) and ML domain-containing protein in *T. papuae*. Therefore, it would be useful to characterize these proteins to understand their functions, as they might be potential drug targets for further anti-trichinella development.

The glycerophospholipid metabolic and phosphatidylinositol dephosphorylation processes could be adopted as pathways for drug design because they contain several proteins that are dissimilar to the equivalent proteins in humans. Two phospholipases, PLB and PLA2, are involved in glycerophospholipid metabolism. Three phosphatases, I145P, INPP5 and 72 kDa inositol polyphosphate 5-phosphatase, play roles in the *T. papuae* phosphatidylinositol dephosphorylation process. PLB has structural features similar to Ntn-hydrolases and it could catalyze the hydrolysis of specific amide bonds of many substrates such as antibiotics and proteins^57^. The PLB contributes to the virulence of many pathogens, for example *Candida albicans*, *Cryptococcus neoformans*, *Aspergillus spp., Legionella pneumophila* and *Pseudomonas aeruginosa*, through host membrane lysis^58,59^,. When triazole, a synthetic phospholipase inhibitor, was administered to mice infected with *C. albicans*, parasite tissue penetration was blocked and host death prevented^60^. In case of PLA2, it is an important energy source enzyme and crucial in the development of *Clonorchis sinensis* mediated hepatic fibrosis^61^. PLA2 has been reported its role on membrane remodelling and vesicle secretion in several organisms for example *P. falciparum*^62^ and *Leishmania amazonensis*^63^. Plasmodial PLA2 involves the egress of merozoites from liver-stage schizonts^64^ and merozoite invasion of red blood cells^58^. According to phospholipase roles in other organisms, *T. papuae* phospholipases are potential targets for further drug development. I145P, INPP5 and 72 kDa inositol polyphosphate 5-phosphatase are related to secondary-messenger-mediating cell responses to various stimulations and regulate Golgi-vesicular trafficking^65^. No studies have been carried out on *T. papuae* I145P, INPP5 or 72 kDa inositol polyphosphate 5-phosphatase in term of their roles and functions. Therefore, it would be useful to characterize these proteins for a better understanding of *T. papuae* lipid metabolic processes.

Our lipidomics study of *T. papuae* larvae provided insights into the lipids and lipid metabolism in the parasites, revealing several potential drug targets which may facilitate the development of innovative parasite control mechanisms. The glycerophospholipid metabolism process and phosphatidylinositol dephosphorylation process are different between *T. papuae* and human. However, the lipidome of *T. spiralis*, an encapsulated *Trichinella* species, will need to be studied and comparatively analysed with that of *T. papuae*. These studies may lead to the development of effective prevention and control strategies against trichinosis caused by both *Trichinella* clades.

## Methods

### *T. papuae* preparation

The *T. papuae* used in this study were laboratory strains maintained in the Department of Helminthology, Faculty of Tropical Medicine, Mahidol University, Thailand. Six-week-old female ICR mice were orally infected with 100 larvae. After 2 months of infection, muscle stage larvae (L1) were obtained from the muscle tissue by pepsin digestion (0.7% pepsin [BDH, UK], 0.7% HCl). All procedures performed on animals in this study were approved by the Faculty of Tropical Medicine Animal Care and Use Committee (FTM-ACUC), Mahidol University. The approval number was FTM-ACUC No. 009/2555. All experiments were performed in accordance with relevant guidelines and regulations

### Lipid extraction

*T. papuae* L1 were quickly frozen in liquid nitrogen and then finely ground in a pestle and mortar. Liquid/liquid extraction was then performed following the Bligh and Dyer method^66^. Briefly, the lipids were extracted from the finely ground L1 by adding 1:1 (v/v) chloroform/methanol and mixed using a vortex. The mixture was then centrifuged at 3,000 × *g* for 10 min to achieve phase separation. The lower chloroform phase was carefully transferred to a separate 1.5-ml tube and dried in a fume hood.

### Ultra-performance liquid chromatography-tandem mass spectrometry (UPLC-MS/MS)

Lipid profile analysis was conducted using ACQUITY UPLC with a 2777 C autosampler (Waters Corp, USA) coupled to a Xevo G2-XS QTof mass spectrometer (Waters, Manchester, UK) with an ESI source^67^. For each sample, triplicate injections were made for each ionization mode. Intact molecular masses and associated fragmentation products were recorded for all compounds using MS, during which retention-time aligned data were collected in two channels simultaneously, i.e., low collision energy for precursor ions and high collision energy for daughter ions, with no precursor ion selection in the quadrupole. A mass range of m/z 100 to 2000 was acquired for lipids. High mass accuracy was maintained by lock mass correction using leucine enkephalin as a reference mass (m/z 556.2771 in ESI+ and m/z 554.2615 in ESI-).

For lipid profiling, the total lipid extracts were solubilized in 2:1:1 (v/v/v) isopropanol/acetonitrile/water (40 µl) and vortex mixed until complete dissolution. The solution was then diluted with mobile phase A at 1:20 dilution, vortex mixed and transferred to a LC-MS sample vial. The samples were kept at 4 °C in the autosampler, 3 µl was injected into the column, and the samples were separated using an ACQUITY UPLC CSH C18 column (2.1 × 100 mm, 1.7 µm; Waters) with a column temperature of 55 °C. Mobile phase A was composed of 60:40 (v/v) acetonitrile/water with 10 mM ammonium formate and 0.1% formic acid. Mobile phase B was composed of 90:10 (v/v) isopropanol/acetonitrile with 10 mM ammonium formate and 0.1% formic acid. The elution gradient was set as follows: 40–43% B (0.0–2.0 min), 43–50% B (2.0–2.1 min), 50–54% B (2.1–12.0 min), 54–70% B (12.0–12.1 min), 70–99% B (12.1–18.0 min), 99–44% B (18.0–18.1 min) and 40% B (18.1–20 min) with a flow rate 400 µl/min. For the ionization source, capillary voltages of 2.0 kV and 1.0 kV were employed for ESI + and ESI-, respectively, and a 30 V cone voltage applied in both modes. The source and desolvation temperatures were set at 120 °C and 400 °C, respectively, and the desolvation flow rate was 950 L/Hr.

For data analysis and identification, the retention time alignment, peak picking and database searching were accomplished using Progenesis QI (version 2.1, Nonlinear Dynamics, Newcastle, UK). Lipids were identified using (i) MetaScope by searching compound features against LIPID MAPS (http://www.lipidmaps.org/) and (ii) LipidBlast (http://fiehnlab.ucdavis.edu/). The search parameter used for precursor ion mass accuracy was set at 5 ppm and the theoretical fragmentation ion mass accuracy was set at 10 ppm. Reported compound identifications were scored and filtered by isotope similarity ≥90%. Identified compounds with abundance >100 and abundance variation among three replicates with <30% coefficient of variation were investigated.

### Bioinformatics analysis

All genes involved in lipid metabolism were gathered using QuickGO, a web-based tool for gene ontology searching. The term GO:0006629 assigns lipid metabolic processes. Gene products belonging to *T. papuae* were collated. The obtained protein sequences were subjected to the Basic Local Alignment Search Tool (BLAST) and used to search the human non-redundant protein, *Trichinella* and *C. elegans* sequence databases. Any proteins with less than a 50% sequence similarity to human proteins were reported as being distinctive *T. papuae* proteins.

## Supplemenatry information

Supplemenatry information.
Supplemenatry dataset 1.
Supplemenatry dataset 2.
