# Supplementary material for: Lipid profile of Trichinella papuae muscle-stage larvae
Source: Sci Rep. 2020 Jun 23;10:10125. doi: 10.1038/s41598-020-67297-8 (PMC7311410; doi:10.1038/s41598-020-67297-8)
Supplement: Supplementary file 1 — Supplemenatry information. [file 41598_2020_67297_MOESM1_ESM.pdf]

Lipid profile of *Trichinella papuae* muscle-stage larvae

Suthee Mangmee[1], Poom Adisakwattana[2], Phornpimon Tipthara[3], Nattapon Simanon[1], Piengchan Sonthayanon[1], Onrapak Reamtong[1]*

[1] Department of Molecular Tropical Medicine and Genetics, Faculty of Tropical Medicine, Mahidol University, Bangkok, 10400, Thailand

[2] Department of Helminthology, Faculty of Tropical Medicine, Mahidol University, Bangkok, 10400, Thailand

[3] Mahidol-Oxford Tropical Medicine Research Unit, Faculty of Tropical Medicine, Mahidol University, Bangkok, 10400, Thailand

*Correspondence to: Onrapak Reamtong, Department of Molecular Tropical Medicine and Genetics, Faculty of Tropical Medicine, Mahidol University, Bangkok, 10400, Thailand

Email: onrapak.rea@mahidol.ac.th

Tel: 66 (0) 2306-9138

Fax: 66 (0) 2306-913

Supplementary Figure 1. Venn diagram of *T. papuae* lipid analysis using negative and positive ESI ionization modes

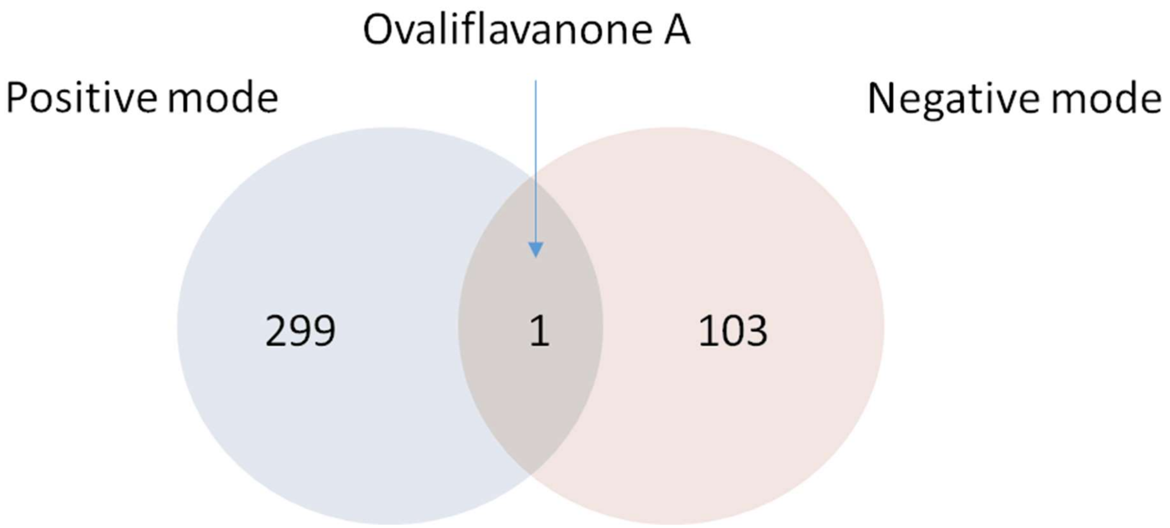

Supplementary Table 1. Top-10 most abundant *T. papuae* lipids in each class

Rank	Average abundance	Group	Description	HMDB
1	28266.02201	Fatty acyl	4,8,16-trimethyldotriacontane	-
2	26816.69162	Fatty acyl	11,21-dimethylheptatriacontane	-
3	15966.52467	Fatty acyl	(9Z)-3-hydroxyoctadecenoylcarnitine	-
4	11669.50986	Fatty acyl	4,5-epoxy-17R-HDHA	-
5	8801.6102	Fatty acyl	N-stearoyl valine	-
6	6720.45933	Fatty acyl	Rosafluine	-
7	6555.183237	Fatty acyl	3-hydroxytetradecanoylcarnitine	+
8	2693.987775	Fatty acyl	O-behenoylcarnitine	+
9	1807.359689	Fatty acyl	Delta2-THA	-
10	1345.005611	Fatty acyl	Anandamide (20:l, n-9)	-
1	1781765.927	Glycerolipid	DG(20:1(11Z)/22:4(7Z,10Z,13Z,16Z)/0:0)[iso2]	+
2	123228.9983	Glycerolipid	DGDG(20:2/14:1)	-
3	70573.87045	Glycerolipid	TG(12:0/13:0/15:1(9Z))[iso6]	-
4	29900.08691	Glycerolipid	DG(16:0/18:0/0:0)[iso2]	+
5	21861.68577	Glycerolipid	DG(9:0/23:0/0:0)	-
6	16720.21503	Glycerolipid	DGDG(23:0/26:0)	-
7	10851.29032	Glycerolipid	DG(18:0/20:4(5Z,8Z,11Z,14Z)/0:0)[iso2]	+
8	10759.69554	Glycerolipid	DG(15:0/21:0/0:0)	+
9	10542.75374	Glycerolipid	DGDG(26:0/25:0)	-
10	7235.251516	Glycerolipid	DG(18:0/22:4(7Z,10Z,13Z,16Z)/0:0)[iso2]	+
1	238658.3382	Glycerophospholipid	PG(21:0/20:0)	-
2	174851.3318	Glycerophospholipid	PC(22:4(7Z,10Z,13Z,16Z)/16:0)	+
3	170915.1218	Glycerophospholipid	PC(16:0/20:4(8Z,11Z,14Z,17Z))	+
4	148731.0248	Glycerophospholipid	PC(18:2(9Z,12Z)/18:0)	+
5	147789.3157	Glycerophospholipid	PC(18:1(11Z)/16:0)	+
6	146510.6256	Glycerophospholipid	PE(18:0/22:4(7Z,10Z,13Z,16Z))	+
7	121459.4376	Glycerophospholipid	PG(13:0/14:0)	-

8	108201.4179	Glycerophospholipid	PC(18:1(15Z)/18:1(15Z))	-
9	91786.21297	Glycerophospholipid	PE(18:2(9Z,12Z)/19:0)	-
10	71340.66089	Glycerophospholipid	PC(22:6(4E,7E,10E,13E,16E,19E)/16:0)	-
1	703.4296874	Polyketide	4,5-Di-O-methyl-8-prenylafzelechin-4beta-ol	-
2	325.2086872	Polyketide	Ovaliflavanone A	-
1	38766.10047	Prenol lipid	35-aminobacteriohopane-32,33,34-triol	-
2	16992.98006	Prenol lipid	Coenzyme Q10	+
3	14648.11552	Prenol lipid	Hemigossypol	-
4	12644.7375	Prenol lipid	Adenosylhopane	-
5	6009.56957	Prenol lipid	2E,6Z-farnesal	-
6	3248.107961	Prenol lipid	(+)-Dysideapalaunic acid	-
7	1104.049712	Prenol lipid	Coenzyme Q9	+
1	1669.935538	Saccharolipid	Butyl 4'-O-butanoyl-6-O-hexadecanoyl-neohesperidoside	-
2	456.1571382	Saccharolipid	DAT(18:0/22:0(2Me[S],4Me[S]))	-
1	118105.5424	Sphingolipid	SM(d18:1/18:0)	+
2	49807.74561	Sphingolipid	SM(d18:1/16:0)	+
3	28329.05466	Sphingolipid	Cer(d14:1/22:0)	-
4	20446.14109	Sphingolipid	GlcCer(d14:2(4E,6E)/18:1(9Z))	-
5	19230.97506	Sphingolipid	PE-Cer(d14:2(4E,6E)/24:0)	-
6	14389.09228	Sphingolipid	PE-Cer(d16:1(4E)/22:1(13Z)(2OH))	-
7	13398.56206	Sphingolipid	SM(d18:1/14:0)	+
8	7419.607785	Sphingolipid	MIPC(t18:0/20:0(2OH))	-
9	7379.148375	Sphingolipid	Cer(d16:1/22:0)	-
10	4923.794577	Sphingolipid	CerP(d15:0/20:0)	-
1	228332.4761	Sterol lipid	Alloavicholic acid	-
2	145267.5569	Sterol lipid	27-Norcholestanehexol	+
3	41004.30962	Sterol lipid	beta-Chlorogenin	-
4	37378.84163	Sterol lipid	12alpha-Hydroxy-3-oxo-5alpha-cholan-24-oic Acid	-
5	15357.69711	Sterol lipid	3beta,6beta-Dihydroxychol-4-en-24-oic Acid	-

6	5014.858006	Sterol lipid	1-Hydroxyprevitamin D3 diacetate	-
7	2174.6779	Sterol lipid	(25S)-5alpha-cholestan-3beta,6alpha,7beta,8beta,15alpha,16beta,26-heptol	-
8	1587.569133	Sterol lipid	Cholesterol glucuronide	+
9	317.0139205	Sterol lipid	20:1-Glc-Campesterol	-
10	287.4243563	Sterol lipid	3-Deoxyvitamin D3	-

Supplementary Table 2. Similarity search results for proteins involved in *T. papuae* lipid metabolic process against *T. spiralis*, *T. britovi*, *T. native*, *T. pseudospiralis*, *C. elegans* and human protein database

Entry	Protein	*T. spiralis*	*T. britovi*	*T. nativa*	*T. pseudospiralis*	*C. elegans*	HUMAN
A0A0V1M465	4-hydroxybutyrate coenzyme A transferase (Fragment)	98.76	99.07	98.75	99.38	65.35	N/A
A0A0V1M4L5	4-hydroxybutyrate coenzyme A transferase	98.76	98.18	98.75	98.64	65.35	N/A
A0A0V1M4U9	4-hydroxybutyrate coenzyme A transferase	98.94	97.74	98.94	98.63	70.39	N/A
A0A0V1MCB5	Uncharacterized protein	98.80	98.80	93.45	99.63	71.49	N/A
A0A0V1MEM4	ML domain-containing protein (Fragment)	74.01	77.30	77.08	82.91	N/A	N/A
A0A0V1MPG5	Proactivator polypeptide	94.30	93.70	88.66	95.46	45.78	40.19
A0A0V1MPH7	Proactivator polypeptide (Fragment)	96.33	96.91	90.80	95.22	45.82	40.44
A0A0V1N1W8	Acyl-CoA synthetase family member 2, mitochondrial (Fragment)	50.45	58.78	65.99	62.87	42.84	40.65
A0A0V1N230	Acyl-CoA synthetase family member 2, mitochondrial (Fragment)	50.28	59.84	67.98	62.81	42.39	40.91
A0A0V1ME09	Type I inositol 1,4,5-trisphosphate 5-phosphatase	72.62	74.60	69.16	77.65	38.82	42.43
A0A0V1MUK0	Nogo-B receptor	98.07	98.06	98.07	96.46	44.62	43.27
A0A0V1MWX4	Ganglioside GM2 activator	81.75	74.11	74.28	64.79	N/A	44.00
A0A0V1N334	Inositol polyphosphate 5-phosphatase K	56.20	60.99	55.19	57.74	41.93	44.68

A0A0V1MHP3	3 beta-hydroxysteroid dehydrogenase/Delta 5-->4-isomerase type 2	99.18	99.45	99.45	98.21	43.44	44.70
A0A0V1N2I4	Inositol polyphosphate 5-phosphatase K (Fragment)	56.58	61.57	55.69	57.73	41.93	44.70
A0A0V1MY16	Group XIIA secretory phospholipase A2	96.67	96.67	96.67	95.04	N/A	44.75
A0A0V1MK15	85/88 kDa calcium-independent phospholipase A2	53.44	48.59	51.80	62.29	48.56	45.40
A0A0V1MQ27	Proactivator polypeptide	88.12	91.83	90.82	85.07	N/A	45.48
A0A0V1N2X4	Inositol polyphosphate 5-phosphatase K	57.47	62.43	56.62	59.26	43.05	45.50
A0A0V1N2G7	Inositol polyphosphate 5-phosphatase K (Fragment)	57.88	63.06	57.18	58.89	43.05	45.54
A0A0V1MA00	GPI ethanolamine phosphate transferase 2	54.29	72.65	60.25	67.70	49.93	45.94
A0A0V1MVT7	85/88 kDa calcium-independent phospholipase A2 (Fragment)	52.14	50.37	57.47	86.02	44.12	46.00
A0A0V1N881	72 kDa inositol polyphosphate 5-phosphatase	54.53	53.42	53.70	60.88	48.20	46.13
A0A0V1N7B3	Acidic phospholipase-like protein	70.17	92.57	62.97	84.43	46.08	46.37
A0A0V1MVD9	85/88 kDa calcium-independent phospholipase A2 (Fragment)	52.93	50.80	58.62	87.89	44.62	46.50
A0A0V1MVJ7	85/88 kDa calcium-independent phospholipase A2 (Fragment)	52.93	50.80	58.47	87.92	44.62	46.50
A0A0V1N8C0	Elongation of very long chain fatty acids protein	75.55	75.75	82.25	83.39	56.29	46.57
A0A0V1MJT7	85/88 kDa calcium-independent phospholipase A2 (Fragment)	52.17	48.29	57.88	63.92	48.38	46.67
A0A0V1MHI7	Farnesyl pyrophosphate synthase 2	69.31	54.93	55.65	73.93	51.71	46.83
A0A0V1N0L0	Type I inositol 3,4-bisphosphate 4-phosphatase (Fragment)	89.49	89.07	88.67	97.48	41.99	47.49
A0A0V1N047	Type I inositol 3,4-bisphosphate 4-phosphatase (Fragment)	88.48	88.07	87.67	96.34	41.52	47.58
A0A0V1NA51	Hormone-sensitive lipase	60.30	61.10	66.82	75.74	50.32	47.74
A0A0V1NAA3	Hormone-sensitive lipase	60.09	60.45	67.29	74.34	53.43	47.97
A0A0V1MSM9	Elongation of very long chain fatty acids protein	76.79	76.80	70.41	68.84	55.58	48.20

A0A0V1M5J6	Phosphatidylinositol 4-phosphate 3-kinase C2 domain-containing subunit beta (Fragment)	55.93	62.46	65.57	75.60	54.56	48.32
A0A0V1M748	Phosphatidylinositol 4-phosphate 3-kinase C2 domain-containing subunit beta (Fragment)	56.49	62.42	65.71	75.93	52.21	48.33
A0A0V1MNQ8	Lipase member H (Fragment)	94.88	96.20	95.41	97.46	N/A	48.62
A0A0V1N9B3	Transcription initiation factor TFIID subunit 5	50.83	50.67	57.09	66.66	46.33	49.04
A0A0V1MNV1	Lipase member H (Fragment)	97.68	96.48	98.28	97.09	N/A	49.22
A0A0V1N0Z3	GPI ethanolamine phosphate transferase 3 (Fragment)	63.54	60.52	67.99	62.68	45.76	49.73
A0A0V1NA68	Phospholipase B-like (Fragment)	97.03	97.92	97.63	96.01	54.27	49.87
A0A0V1M5Z1	Phosphatidylinositol 4-phosphate 3-kinase C2 domain-containing subunit alpha (Fragment)	54.06	62.55	63.93	78.07	51.32	50.73
A0A0V1M648	Phosphatidylinositol 4-phosphate 3-kinase C2 domain-containing subunit alpha (Fragment)	54.30	62.77	64.15	78.65	51.57	50.95
A0A0V1N6Z6	Sn1-specific diacylglycerol lipase beta	96.60	96.89	96.52	97.69	51.11	51.23
A0A0V1N0L2	Decaprenyl-diphosphate synthase subunit 1 (Fragment)	44.00	44.82	56.03	50.68	60.38	51.28
A0A0V1N5D5	GPI-anchor transamidase (Fragment)	57.45	59.18	55.78	63.92	54.04	51.49
A0A0V1MS54	Mannosyltransferase	72.18	63.88	57.34	75.49	49.55	51.56
A0A0V1NA66	Hormone-sensitive lipase	94.23	95.79	93.49	94.30	47.29	51.66
A0A0V1MEJ4	Phosphoinositide phospholipase C	62.16	57.60	59.57	79.21	52.38	51.68
A0A0V1MPD7	3'(2'),5'-bisphosphate nucleotidase 1	62.76	65.38	66.92	68.48	51.45	51.87
A0A0V1MQ86	3'(2'),5'-bisphosphate nucleotidase 1	62.76	65.38	66.90	68.50	51.45	51.87
A0A0V1MFB7	Phosphoinositide phospholipase C (Fragment)	61.01	56.95	61.34	76.84	52.41	51.90
A0A0V1MEJ7	Phosphoinositide phospholipase C (Fragment)	60.66	56.37	61.46	76.59	50.77	52.04
A0A0V1N9D1	Phosphatidylinositol 3-kinase catalytic subunit type 3 (Fragment)	47.77	50.18	53.34	67.76	46.62	52.13
A0A0V1MLW1	Phospholipase A(2)	72.24	49.52	84.11	60.74	53.52	52.22
A0A0V1M757	GPI mannosyltransferase 2	80.00	84.22	N/A	96.59	41.28	52.90
A0A0V1MFZ1	Cathepsin B-like cysteine proteinase	70.22	55.25	70.24	76.71	56.01	52.97
A0A0V1MFP3	Cathepsin B-like cysteine proteinase	70.22	55.25	70.24	76.69	56.01	53.00

A0A0V1N8P1	Protein dif-1	52.39	45.90	59.90	73.27	47.64	53.25
A0A0V1N0R7	Phosphoinositide phospholipase C (Fragment)	66.60	55.09	60.07	72.19	49.38	53.57
A0A0V1MF23	Inositol polyphosphate 5-phosphatase OCRL-1 (Fragment)	49.83	46.64	58.17	65.84	46.72	53.72
A0A0V1N288	Polyprenol reductase (Fragment)	93.00	96.55	96.24	93.60	48.39	53.76
A0A0V1MPW0	GPI transamidase component PIG-T	97.40	N/A	95.93	96.87	45.40	53.91
A0A0V1MC98	GPI mannosyltransferase 1 (Fragment)	93.28	92.53	92.77	98.17	57.46	53.92
A0A0V1MG61	Alpha-1,3-glucosyltransferase	67.43	79.19	75.50	82.06	51.07	54.15
A0A0V1N581	Glycerophosphodiester phosphodiesterase 1 (Fragment)	56.22	62.32	64.86	71.97	47.81	54.27
A0A0V1M7J5	1-phosphatidylinositol 3-phosphate 5-kinase	56.02	57.53	60.30	90.41	54.24	54.33
A0A0V1MDW2	Putative glycerophosphocholine phosphodiesterase GPCPD1-like protein T05H10.7 (Fragment)	70.11	62.97	67.58	62.60	45.98	54.33
A0A0V1MLP7	Phosphatidylinositol glycan anchor biosynthesis class U protein (Fragment)	73.26	83.87	81.80	86.75	46.02	54.33
A0A0V1MIX7	Tafazzin family protein (Fragment)	97.96	N/A	94.36	96.71	57.02	54.38
A0A0V1MZQ3	Dol-P-Glc:Glc(2)Man(9)GlcNAc(2)-PP-Dol alpha-1,2-glucosyltransferase	96.76	95.46	95.25	96.54	48.97	54.62
A0A0V1N9A5	Protein dif-1	49.76	46.17	58.74	75.13	49.90	54.65
A0A0V1MW22	Sphingomyelin phosphodiesterase	96.56	96.80	91.76	98.62	52.56	54.68
A0A0V1N8U0	Protein dif-1	49.01	46.24	58.09	74.04	49.38	54.90
A0A0V1MWW9	ATP-citrate synthase	86.68	74.97	67.61	97.98	85.89	54.92
A0A0V1NAH6	Choline/ethanolamine kinase (Fragment)	95.48	97.23	97.81	98.90	51.55	54.92
A0A0V1N9X6	Choline/ethanolamine kinase	96.55	98.41	98.08	99.04	51.70	54.94
A0A0V1MGS2	Alpha-1,3-glucosyltransferase	69.87	80.10	75.37	85.89	54.56	55.24
A0A0V1N8G0	Protein dif-1	49.75	46.12	58.49	74.86	49.95	55.29
A0A0V1MYJ1	Decaprenyl-diphosphate synthase subunit 2	90.56	90.36	90.05	96.79	48.34	55.35
A0A0V1N2S1	1-alkyl-2-acetylglycerophosphocholine esterase	92.11	98.46	92.09	98.46	53.72	55.35
A0A0V1MU44	Choline/ethanolaminephosphotransferase 1	95.65	92.94	95.46	94.42	55.07	55.60

A0A0V1MGI3	Type I inositol 1,4,5-trisphosphate 5-phosphatase	69.26	69.08	75.26	67.06	45.82	55.62
A0A0V1MW19	Sphingomyelin phosphodiesterase	96.56	96.76	91.88	98.57	53.09	55.67
A0A0V1M5K3	Patatin-like phospholipase domain-containing protein 4	76.52	62.91	76.15	59.55	57.29	55.80
A0A0V1N2E3	Phosphodiesterase (Fragment)	61.23	67.41	64.18	93.54	50.16	56.03
A0A0V1N7D1	PI-PLC X domain-containing protein 3	96.46	95.51	95.82	99.35	47.99	56.04
A0A0V1N7L9	PI-PLC X domain-containing protein 3	96.46	95.51	95.82	99.34	47.99	56.05
A0A0V1N7C3	PI-PLC X domain-containing protein 3	96.09	95.51	96.12	97.33	48.15	56.09
A0A0V1MBQ6	Trifunctional enzyme subunit alpha, mitochondrial	66.31	64.91	60.72	93.15	54.03	56.17
A0A0V1MBW8	Trifunctional enzyme subunit alpha, mitochondrial	66.54	65.28	60.91	93.31	54.01	56.19
A0A0V1MMS7	Phosphatidylinositol 5-phosphate 4-kinase type-2 beta	57.84	67.42	73.43	71.97	54.85	56.33
A0A0V1N0L4	Phosphoinositide phospholipase C (Fragment)	56.40	67.46	70.56	66.68	67.00	56.34
A0A0V1N262	Phosphodiesterase (Fragment)	61.34	67.94	64.31	92.95	50.19	56.34
A0A0V1N273	Phosphodiesterase (Fragment)	61.37	67.98	64.02	93.82	50.19	56.34
A0A0V1MMH8	Phosphatidylinositol glycan anchor biosynthesis class U protein (Fragment)	79.27	94.93	94.81	88.27	49.01	56.65
A0A0V1N151	Phosphoinositide phospholipase C (Fragment)	56.64	67.61	70.75	67.17	67.54	56.65
A0A0V1MBH4	Trifunctional enzyme subunit alpha, mitochondrial	65.02	64.70	59.61	91.58	53.57	56.71
A0A0V1N9E9	Lipase	62.61	63.53	69.57	60.30	52.50	56.79
A0A0V1M5U8	Trifunctional enzyme subunit alpha, mitochondrial (Fragment)	67.28	66.88	60.69	94.08	53.88	56.80
A0A0V1MBB1	Trifunctional enzyme subunit alpha, mitochondrial	66.03	64.40	60.33	92.10	53.54	56.91
A0A0V1M5W2	Trifunctional enzyme subunit alpha, mitochondrial (Fragment)	67.42	66.97	60.83	94.14	54.20	57.06
A0A0V1MMS5	Phosphatidylinositol 5-phosphate 4-kinase type-2 beta	57.08	60.07	59.93	62.95	54.00	57.41

A0A0V1MBJ0	Trifunctional enzyme subunit alpha, mitochondrial	65.91	64.00	60.39	91.89	53.42	57.50
A0A0V1MIB6	Diphosphomevalonate decarboxylase	96.17	96.73	96.73	96.80	59.38	57.80
A0A0V1MI92	Palmitoyltransferase	55.97	53.21	57.97	63.34	50.48	57.95
A0A0V1MD36	Squamous cell carcinoma antigen recognized by T-cells 3 (Fragment)	51.72	57.98	62.64	54.19	48.82	58.11
A0A0V1M4M0	Phosphoinositide phospholipase C	60.96	65.87	60.26	76.62	54.29	58.38
A0A0V1MVJ2	Synaptojanin-1	52.82	51.01	53.91	68.35	47.03	58.38
A0A0V1MIB5	Phosphoinositide phospholipase C	58.69	58.00	70.48	68.03	50.65	58.43
A0A0V1MIQ8	Phosphoinositide phospholipase C	58.71	58.02	70.51	68.07	50.63	58.43
A0A0V1MGL3	Lipase	74.76	75.82	68.11	80.90	53.04	58.45
A0A0V1MVG3	Synaptojanin-1	52.14	50.32	53.67	68.03	46.70	58.61
A0A0V1MGX1	CDP-diacylglycerol--glycerol-3-phosphate 3-phosphatidyltransferase	95.03	95.13	95.05	96.74	61.52	58.75
A0A0V1N7E4	PI-PLC X domain-containing protein 3	93.20	91.59	85.17	96.23	53.47	58.83
A0A0V1MNG5	Phosphatidylinositol 5-phosphate 4-kinase type-2 beta	59.66	62.57	61.42	65.54	56.69	58.84
A0A0V1MMR7	Phosphatidylinositol 5-phosphate 4-kinase type-2 beta	59.74	62.59	61.31	65.45	56.69	58.85
A0A0V1MGA5	CDP-diacylglycerol--glycerol-3-phosphate 3-phosphatidyltransferase	95.12	94.90	95.13	96.41	62.25	58.97
A0A0V1MGD4	Lipase	74.30	75.69	67.77	80.31	52.56	59.05
A0A0V1MZX1	Putative endochitinase	55.76	60.36	69.01	67.44	46.14	59.05
A0A0V1N702	Myotubularin-related protein 6	58.12	61.80	63.88	78.88	50.94	59.15
A0A0V1MVM3	GPI ethanolamine phosphate transferase 1	56.33	49.42	53.95	76.16	46.18	59.35
A0A0V1M954	Protein VAC14-like protein	98.57	97.43	94.62	97.87	61.79	59.47
A0A0V1N0Q8	Mevalonate kinase	81.87	96.57	95.99	96.58	69.79	59.70
A0A0V1N0K3	Mevalonate kinase (Fragment)	97.22	96.57	95.96	97.98	69.79	59.78
A0A0V1MIT0	Putative 3-hydroxyacyl-CoA dehydrogenase	61.17	65.14	68.57	62.57	58.60	59.81
A0A0V1MUS1	Choline/ethanolaminephosphotransferase 1	95.86	98.71	95.54	97.37	58.16	59.85
A0A0V1MWH5	Fatty acid 2-hydroxylase	99.35	99.35	95.32	98.82	63.04	59.87

A0A0V1N0C7	Putative endochitinase	55.76	60.59	69.63	68.19	46.45	60.03
A0A0V1M491	Very-long-chain enoyl-CoA reductase	95.98	97.33	94.31	95.69	51.12	60.10
A0A0V1N6Z8	Myotubularin-related protein 6	59.42	62.94	64.91	79.60	52.63	60.19
A0A0V1M7D2	Phosphatidylinositol 4-kinase type 2-beta (Fragment)	77.50	80.06	75.56	71.81	53.95	60.47
A0A0V1M7E2	Phosphatidylinositol 4-kinase type 2-beta (Fragment)	77.34	80.04	75.48	71.80	53.88	60.47
A0A0V1MK44	Phosphatidylinositol 4-kinase type 2-beta	77.68	79.02	76.22	72.42	53.60	60.47
A0A0V1MYB0	Glucosylceramidase	96.24	98.17	96.12	93.79	61.61	60.60
A0A0V1N0M2	Mevalonate kinase (Fragment)	96.71	95.80	94.41	98.37	71.43	60.66
A0A0V1MQ99	Mitochondrial translocator assembly and maintenance protein 41-like protein (Fragment)	94.66	91.40	91.18	94.68	57.50	60.91
A0A0V1MQP5	Phosphatidylinositol 4-kinase beta	57.54	53.38	68.07	71.28	52.60	61.12
A0A0V1MQJ2	Mitochondrial translocator assembly and maintenance protein 41-like protein (Fragment)	96.14	95.07	95.07	97.78	61.03	61.28
A0A0V1MCE4	Phosphatidylinositol 3-kinase age-1	56.16	54.59	57.45	67.40	49.18	61.40
A0A0V1MB02	3-hydroxy-3-methylglutaryl coenzyme A synthase	96.06	96.27	96.47	99.17	59.26	61.45
A0A0V1M5K5	Serine incorporator 1 (Fragment)	90.04	90.13	91.72	96.44	65.01	61.77
A0A0V1M5L2	Serine incorporator 1 (Fragment)	88.48	88.57	90.27	95.38	63.97	61.78
A0A0V1MPL8	Spectrin alpha chain	64.14	68.26	79.32	91.14	53.19	61.79
A0A0V1MQ97	Mitochondrial translocator assembly and maintenance protein 41-like protein (Fragment)	87.47	84.62	84.16	89.75	61.03	61.82
A0A0V1M9C3	Phospholipase D1 (Fragment)	89.51	90.63	89.63	93.51	68.08	61.96
A0A0V1MLF3	U4/U6 small nuclear ribonucleoprotein Prp31 (Fragment)	54.87	59.24	58.13	62.95	51.38	62.22
A0A0V1MKT1	Inositol-1-monophosphatase	70.21	64.87	59.21	53.23	46.41	62.28
A0A0V1MB87	Phosphatidylinositol 4-phosphate 5-kinase type-1 alpha (Fragment)	75.50	60.94	52.17	55.86	56.30	62.88
A0A0V1N1F4	Isopentenyl-diphosphate Delta-isomerase II (Fragment)	94.93	85.71	87.77	93.40	55.67	63.04
A0A0V1M528	Serine incorporator 1 (Fragment)	91.66	91.74	93.23	98.45	66.11	63.39
A0A0V1MHE6	Group 3 secretory phospholipase A2	94.74	94.74	96.58	98.44	N/A	63.56

A0A0V1MWG6	Phosphatidylserine synthase 1	91.45	97.36	98.29	95.65	70.10	63.88
A0A0V1MX28	Phosphatidylserine synthase 1 (Fragment)	91.47	97.27	98.29	95.65	70.10	63.88
A0A0V1MUG7	High-affinity choline transporter 1	57.53	60.31	62.40	71.91	51.39	64.06
A0A0V1MUS4	High-affinity choline transporter 1	77.23	72.36	73.15	75.50	58.29	64.06
A0A0V1N8X0	PNPLA domain-containing protein	76.39	86.82	75.42	91.70	50.97	64.18
A0A0V1MJ16	Putative 3-hydroxyacyl-CoA dehydrogenase	64.09	62.71	68.21	63.26	56.90	64.56
A0A0V1M8K9	Neuropathy target esterase sws	94.26	94.99	93.55	96.05	57.02	64.78
A0A0V1N886	PNPLA domain-containing protein	75.94	89.03	75.30	93.15	51.68	64.80
A0A0V1N8Y4	PNPLA domain-containing protein	76.12	89.10	75.53	92.92	51.50	64.80
A0A0V1MH50	Alpha-1,3-glucosyltransferase	74.37	66.58	68.83	70.65	59.50	65.02
A0A0V1M939	Neuropathy target esterase sws	93.81	94.62	93.52	95.69	56.82	65.07
A0A0V1N7L8	CDP-diacylglycerol--inositol 3-phosphatidyltransferase	95.91	92.51	96.36	94.55	65.42	66.57
A0A0V1M9V8	Neuropathy target esterase	91.59	92.80	92.63	94.79	58.00	66.71
A0A0V1M4T7	Acid ceramidase	96.07	94.47	95.48	98.00	58.64	67.25
A0A0V1M4Z9	Acid ceramidase (Fragment)	96.03	94.42	95.44	98.00	58.69	67.25
A0A0V1M504	Acid ceramidase (Fragment)	95.83	94.35	95.20	98.00	58.69	67.25
A0A0V1MVQ5	Histone-lysine N-methyltransferase	55.84	61.52	79.85	84.27	50.95	67.85
A0A0V1MJJ1	Hydroxyacyl-coenzyme A dehydrogenase, mitochondrial (Fragment)	63.29	61.62	69.86	64.25	58.18	67.93
A0A0V1MV43	Histone-lysine N-methyltransferase	56.02	61.44	80.12	84.38	50.98	67.93
A0A0V1MXQ1	3-hydroxy-3-methylglutaryl-coenzyme A reductase	91.88	92.86	92.56	93.69	63.41	68.00
A0A0V1MHH9	Alpha-1,3-glucosyltransferase (Fragment)	73.43	65.62	63.54	70.41	60.64	68.12
A0A0V1MW15	Putative cardiolipin synthase 1	95.31	95.35	95.33	98.44	59.84	68.16
A0A0V1MHZ4	Acetyl-CoA carboxylase (Fragment)	52.85	58.09	53.96	73.26	52.46	68.17
A0A0V1MIR2	Acetyl-CoA carboxylase	52.85	58.18	54.04	73.01	52.45	68.19
A0A0V1N794	ORM1-like protein 3 (Fragment)	99.35	99.35	100.00	98.48	N/A	68.34
A0A0V1MT16	N-acetylglucosaminyl-phosphatidylinositol de-N-acetylase	97.44	98.90	98.41	99.02	51.61	69.48

A0A0V1MXT6	3-hydroxy-3-methylglutaryl coenzyme A reductase	95.00	98.27	95.81	97.63	66.34	69.51
A0A0V1MXU7	3-hydroxy-3-methylglutaryl coenzyme A reductase	94.66	98.28	95.83	98.00	66.34	69.51
A0A0V1MW14	Phosphatidate cytidylyltransferase	98.89	99.39	98.93	94.98	72.67	69.68
A0A0V1N0A8	Phosphatidylinositol 4-kinase type 2-beta	64.53	76.66	67.33	79.85	61.67	70.50
A0A0V1N0A9	Phosphatidylinositol 4-kinase type 2-beta	65.29	74.82	67.25	82.04	61.67	70.50
A0A0V1MSN3	Ankyrin-2	54.50	59.05	78.14	87.16	70.04	71.70
A0A0V1MRV3	Ankyrin-2	54.52	59.05	77.90	87.37	70.04	72.20
A0A0V1MCF8	Putative sphingolipid delta(4)-desaturase/C4-hydroxylase (Fragment)	96.82	97.85	97.90	96.99	74.17	72.24
A0A0V1M968	Lipoyl synthase, mitochondrial	92.41	96.38	96.92	98.24	75.08	73.12
A0A0V1MRL4	Acyl carrier protein	84.40	90.51	83.40	90.09	54.15	73.35
A0A0V1MY09	3-hydroxy-3-methylglutaryl coenzyme A reductase (Fragment)	98.79	99.07	98.55	98.91	67.92	73.65
A0A0V1MXB8	3-hydroxy-3-methylglutaryl-coenzyme A reductase (Fragment)	99.63	99.63	99.26	99.57	66.16	76.65
A0A0V1MM53	Succinate dehydrogenase [ubiquinone] flavoprotein subunit, mitochondrial	74.71	61.84	70.79	70.85	76.51	76.87
A0A0V1N9V1	Acyl carrier protein (Fragment)	81.45	80.63	83.96	81.80	69.87	79.55
A0A0V1N184	60S ribosomal protein L27a (Fragment)	96.38	80.10	83.11	94.73	73.34	81.83
